# Structural Remodeling and Rotational Activity in Persistent/Long-Lasting Atrial Fibrillation: Gender-Effect Differences and Impact on Post-ablation Outcome

**DOI:** 10.3389/fcvm.2022.819429

**Published:** 2022-03-21

**Authors:** Gonzalo R. Ríos-Muñoz, Nina Soto, Pablo Ávila, Alejandro Carta, Felipe Atienza, Tomás Datino, Esteban González-Torrecilla, Francisco Fernández-Avilés, Ángel Arenal

**Affiliations:** ^1^Department of Cardiology, Instituto de Investigación Sanitaria Gregorio Marañón (IiSGM), Hospital General Universitario Gregorio Marañón, Madrid, Spain; ^2^Center for Biomedical Research in Cardiovascular Disease Network (CIBERCV), Madrid, Spain; ^3^Departamento de Bioingeniería e Ingeniería Aeroespacial, Universidad Carlos III de Madrid, Madrid, Spain; ^4^Departament de Medicina, Universitat Autònoma de Barcelona, Barcelona, Spain; ^5^Facultad de Medicina, Universidad Complutense de Madrid, Madrid, Spain

**Keywords:** atrial fibrillation, gender, rotational activity, ultra-high density mapping, AF ablation

## Abstract

**Background:**

Structural and post-ablation gender differences are reported in atrial fibrillation (AF). We analyzed the gender differences in structural remodeling and AF mechanisms in patients with persistent/long-lasting AF who underwent wide area circumferential pulmonary vein isolation (WACPVI).

**Materials and Methods:**

Ultra-high-density mapping was used to study atrial remodeling and AF drivers in 85 consecutive patients. Focal and rotational activity (RAc) were identified with the CartoFinder system and activation sequence analysis. The impact of RAc location on post-ablation outcomes was analyzed.

**Results:**

This study included 64 men and 21 women. RAc was detected in 73.4% of men and 38.1% of women (*p* = 0.003). RAc patients had higher left atrium (LA) voltage (0.64 ± 0.3 vs. 0.50 ± 0.2 mV; *p* = 0.01), RAc sites had higher voltage than non-RAc sites 0.77 ± 0.46 vs. 0.53 ± 0.37 mV (*p* < 0.001). Women had lower LA voltage than men (0.42 vs. 0.64 mV; *p* < 0.001), including pulmonary vein (PV) antra (0.16 vs. 0.30 mV; *p* < 0.001) and posterior wall (0.34 vs. 0.51 mV; *p* < 0.001). RAc in the posterior atrium was recorded in few women (23.8 vs. 54.7% in men; *p* = 0.014). AF recurrence rate was higher in patients with RAc outside WACPVI than those with all RAc inside WACPVI or no RAc (63.4 vs. 11.1 and 31.0%; *p* = 0.008 and *p* = 0.01). Comparison of selected patients using propensity score matching confirmed lower atrial voltage (0.4 ± 0.2 vs. 0.7 ± 0.3 mV; *p* = 0.007) and less RAc (38 vs. 75%; *p* = 0.02) in women.

**Conclusion:**

Women have shown more advanced structural remodeling at ablation, which is associated with a lower incidence of RAc (usually located outside the WACPVI). These findings could explain post-ablation gender differences.

## Introduction

Recent studies have reported the presence of multiple gender-related differences in patients with atrial fibrillation (AF) (1, 2). Women are older at the moment of the procedure and the probability of recurrences after catheter ablation (3) is significantly higher (4, 5). A recent study reported that after multiple procedures, women were more likely to have recurrences, despite that pulmonary veins (PVs) reconnection was less frequent (6). In long-standing AF, female sex was an independent factor for presenting a higher degree of fibrosis in the atria (7). The magnitude of this problem has been recently highlighted. At 30 days after ablation, readmissions were 48% higher in women than men, and the female sex was independently associated with readmission for AF/atrial tachycardia (AT) (8). These studies suggest that the structural remodeling and AF drivers could be different in women.

Multiple wandering wavelets and independent drivers have been proposed as the mechanisms maintaining AF (9–11), with studies ablating possible AF drivers outside PVs showing promising results (12, 13). Alleged AF drivers can be identified by wavefronts emanating from focal sources (foci or breakthrough sites) or by rotational activity (RAc) (11, 14–16).

Ultra-high-density mapping and new tools for the detection of AF drivers (17–19), may better define the structural remodeling and the mechanisms maintaining persistent AF. We evaluated the relationship between AF drivers and structural remodeling and the gender effect. Additionally, the present study evaluated the effect of the presence and anatomical distribution of alleged AF drivers on AF recurrences after wide area circumferential pulmonary vein isolation (WACPVI).

## Materials and Methods

### Study Population

We studied a retrospective cohort of 90 (66 men and 23 women) consecutive patients with long-standing/persistent AF who underwent ultra-high-density mapping during stable AF and point-by-point WACPVI (16 redo procedures; 13 men, 3 women; *p* = 0.542). In 67 patients, AF was present at the arrival and at the electrophysiology laboratory, and in 18 patients it was induced by rapid pacing and remained stable thereafter. In 4 patients (2 men and 2 women) AF could not be induced and therefore they were excluded since RAc mapping could not be performed. The final sample size of the study was 85 patients (64 men and 21 women). AF terminated (sinus rhythm or AT) in 10 patients. The study was approved by the ethics committee of the center.

### Electrophysiological Study and Electroanatomical Mapping

The electrophysiology study was performed under general anesthesia. Through the femoral vein, one decapolar catheter was placed in the coronary sinus and transseptal puncture was performed to advance the mapping and ablation catheters to the left atrium (LA). All patients underwent electroanatomical (EA) mapping during sustained AF (20). The EA mapping and signal acquisitions were performed using a 20-pole multi-electrode catheter (PentaRay, Biosense Webster, Diamond Bar, CA, USA) and tissue proximity index (TPI) active during all the procedures. For the ablation, an irrigated contact-force sensing catheter was used (SmartTouch, or SmartTouch SF, Biosense Webster, Diamond Bar, CA, USA). The ablation targeted a wide area around the PVs antra to achieve pulmonary vein isolation (PVI); additional lesions were delivered at the carinas. Ablation lesions were guided by the Ablation Index (CARTO3 V7, Biosense Webster, Diamond Bar, CA, USA), targeting values of 350 or 450 units at the posterior or anterior left atrial wall, respectively (21). Radiofrequency power was set at 35–50 W at the anterior aspect of the veins and 25–45 W at the posterior aspect, with an irrigation flow of 30 ml/min using power control mode.

### Rotational and Focal Activity Mapping

**Figure 1:**
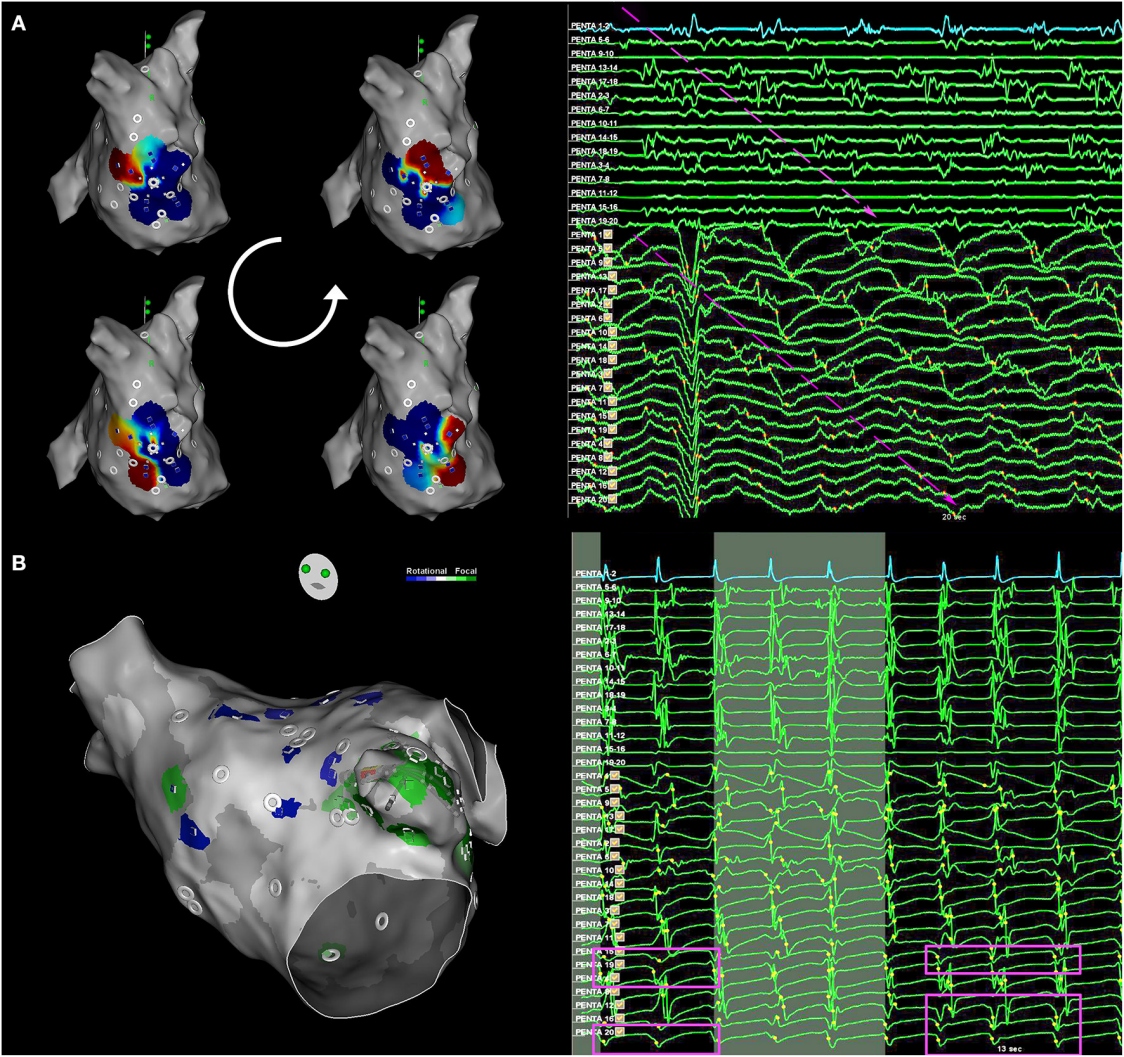
Rotational and focal activity detection. **(A)** Rotational activity (RAc) detection with CartoFinder. Left, local activation map interactive view. One cycle of RAc is represented. Right, bipolar and unipolar EGMs from the multi-electrode catheter are shown. Electrograms (EGMs) are ordered by rings. Bipolar EGMs Penta 1–2 to Penta 17–18 form the external ring, Penta 2–3 to Penta 18–19 the middle ring, and Penta 3–4 to Penta 19-20 the internal ring. Unipolar EGMs Penta 1–17 form the external ring, Penta 2–18 form the external middle ring, Penta 3–19 form the internal middle ring, and Penta 4–20 form the internal ring. The staircase activation pattern expands the complete cycle. Long duration and low-amplitude EGMs are recorded by nearby electrodes: Penta 15–16, Penta 19–20, and Penta 18–19, where the center of rotation is located. The RAc has been highlighted by arrows. EGMs, electrograms. **(B)** Focal activity detection with CartoFinder. Left, the electroanatomical (EA) map view of the catheter was placed on the left atrial appendage. Focal activation is highlighted in green. Right, the darkest background window intervals show when repetitive focal activation events are detected with CartoFinder. The QS patterns are highlighted. Bipolar and unipolar EGMs are shown.

The CartoFinder module in the CARTO3 V7 system detected rotational and focal activity (FAc) from unipolar electrogram (EGM) activation patterns (17–19). The module uses the PentaRay EGMs and acquires windows of 30 s for an activation sequence analysis (Figure 1). Acquisitions were accepted when the catheter splines were orderly deployed and in contact with the atrial wall. Briefly, the module detects the local activation times (LATs) as the time instant of the maximum negative slope of unipolar EGMs. The CartoFinder module detects an RAc event if the LATs of the four concentric rings span >50% of the dominant cycle length of the unipolar EGMs for two or more consecutive beats (rotations). The acquisition point and area covered by the catheter are highlighted on the CARTO3 3D mesh as a region of interest if a repetitive RAc is detected. Bipolar EGMs of all acquisitions in which the CartoFinder detected RAc were reviewed by expert electrophysiologists and the presence of RAc was only confirmed if there were more than 2 rotations with staircase activation sequence and simultaneous acceleration of discrete EGMs and appearance of continuous activity (22) (Figure 1A).

Moreover, CartoFinder provided FAc assessment. The algorithm identifies unipolar EGMs that exhibit a QS pattern. The module identifies the earliest QS morphology within a 10 mm radius and 50 ms window before its annotation. If the QS pattern occurs for two or more consecutive beats, it is considered repetitive (Figure 1B).

The continuous electrical activity that is a footprint of RAc (22) was quantified in each CartoFinder acquisition. The electrical burden of the bipolar EGMs was measured using an automatic activity detection algorithm based on the fully unsupervised Hidden Markov Models (HMMs) (23). The method automatically detects periods of electrical activity in AF bipolar EGMs and provides a score between 0 (no activity) and 1 (continuous activity), as shown in the Supplementary Material.

### Mapping Data Analysis

The EA maps and CartoFinder files (3D coordinates and EGMs signals) were exported for offline processing and analysis. The maps were merged into a reference LA mesh for analysis with an in-house algorithm (Supplementary Figure 1). A 3D atrial shell with well-defined PVs and LAA was selected as the reference. The 3D meshes were pre-aligned by centering them at point [0, 0, 0] in the 3D axes. Then, the algorithm applied a non-rigid Iterative Closest Point (ICP) method to merge all atrial meshes into the reference one (24). The algorithm automatically projected the voltage, cycle length, and driver locations onto the reference mesh. This merging method preserves the atrial structures and anatomical information and avoids manual quantification or visual transformations, e.g., representing the LA 3D mesh with a 2D projection.

The 3D left atrial shell was manually segmented into 9 areas: left atrial appendage (LAA), PVs, posterior wall, atrial roof, lateral wall, septum, anterior wall, atrial floor, and mitral ring. The PV antra limits were established at 1 cm of the PV ostia but at the anterior aspect of the left superior PV, in which the antrum limit was the ridge between the vein and the LAA (Supplementary Figure 2). The EA area and volume of the left atrium was obtained from the CARTO3 software excluding the PVs and mitral valve that were manually removed.

All the EA maps were reviewed to locate the RAc events with respect to the PV isolation lines by an author blinded to the recurrence status.

### Discharge and Follow-Up

Antiarrhythmic drugs were maintained for 3 months and anticoagulation therapy for 8 weeks and thereafter it was based on the CHA2DS2-VASc score. Outpatient clinical follow-up was scheduled at 3, 6, and 12 months and included an ECG and a 24-h Holter recording. Any symptomatic or documented episodes of AF/AT longer than 30 s were considered as recurrence.

### Statistical Analysis

We present categorical values as absolute and relative percentages (frequencies), and normally distributed variables are summarized by the mean and SD. We tested categorical data with the chi-square test, continuous variables using Welch's two-sample *t*-test, and proportions based on normal *z*-test. Contingency tables were analyzed using the chi-tests or Fisher's exact tests when appropriate. A logistic regression propensity score matching was applied to reduce the effect of baseline differences in the data population by gender, Supplementary Table 4. The propensity score analysis is detailed in the Supplementary Material. The analysis was performed in Python (PyCharm 2020.1.3) and JMP statistical software (14.3.0). All tests were 2-tailed, and a *p* < 0.05 was considered statistically significant.

## Results

### Patient Demographic Characteristics

A total of 85 (64 men and 21 women) persistent/long-standing patients with AF were included in the study. Baseline characteristics are shown in Table 1. The AF duration was 3.2 ± 3.8 years for men and 2.4 ± 2.9 for women (*p* = 0.284). A left ventricular ejection fraction (LVEF) <50% was observed in 16 of the patients. Overall, women were older, had a higher CHA2DS2VASc score (*p* < 0.001), and a higher prevalence of hypertension (*p* = 0.028). These values and the BSA were used as covariates to perform the propensity score matching technique. Supplementary Table 1 includes the paired baseline analysis by gender with no significant differences.

**Table 1:** Baseline characteristics of the patients. Rotational activity (RAc) and gender differences.

		**Overall**	**Men**	**Women**	***P*** -value^*^
N		85 (100.0)	64 (75.3)	21 (24.7)	-
Age (years)		60.9 ± 9.5	59.5 ± 9.3	65.2 ± 8.8	**0.014**
Procedure number		1.2 ± 0.5	1.2 ± 0.5	1.2 ± 0.5	0.823
**Comorbidities**					
Heart failure		13 (15.3)	13 (20.3)	4 (19.0)	0.897
Hypertension		41 (48.2)	26 (40.6)	15 (71.4)	**0.028**
Diabetes mellitus		17 (20.0)	11 (17.2)	6 (28.6)	0.345
Dyslipidemia		30 (35.3)	20 (31.2)	10 (47.6)	0.272
COPD		5 (5.9)	5 (7.8)	0 (0.0)	0.326
Obstructive sleep apnea		17 (20.0)	15 (23.4)	2 (9.5)	0.219
Stroke		7 (8.2)	5 (7.8)	2 (9.5)	0.803
SHD		28 (32.9)	23 (35.9)	5 (23.8)	0.448
BSA (*m*^2^)		2.0 ± 0.2	2.1 ± 0.2	1.8 ± 0.1	**<0.001**
CHA2DS2-VASc		1.9 ± 1.5	1.5 ± 1.3	3.1 ± 1.3	**<0.001**
New York heart association functional classification		9 (10.6)	6 (9.4)	3 (14.3)	0.186
	I	28 (32.9)	25 (39.1)	3 (14.3)	
	II	30 (35.3)	22 (34.4)	8 (38.1)	
	III	17 (20.0)	10 (15.6)	7 (33.3)	
	IV	1 (1.2)	1 (1.6)		
Diagnosis of AF (years)		3.0 (3.6)	3.2 (3.8)	2.4 (2.9)	0.284
**Echocardiographic parameters**					
LVEF (%)		55.6 ± 10.8	55.5 ± 10.3	55.7 ± 12.5	0.953
LA area (*cm*^2^)		29.3 ± 23.9	30.7 ± 27.1	24.9 ± 5.1	0.176
LA area/BSA (*cm*^2^/*m*^2^)		14.7 ± 11.6	14.9 ± 13.1	13.9 ± 2.9	0.477

*Values in the table are n (%) or mean ± SD. COPD, chronic obstructive pulmonary disease; SHD, structural heart disease; BSA, body surface area; NYHA, New York Heart Association; LVEF, left ventricular ejection fraction; and LA, left atrium*.

* *Categorical data with the chi-square test for categorical data, continuous variables using Welch's two-sample t-test, and proportions based on normal z-test. Bold values indicates statistical significance*.

### EA and AF Drivers Mapping

#### Differences Related to RAc and Gender

**Figure 2:**
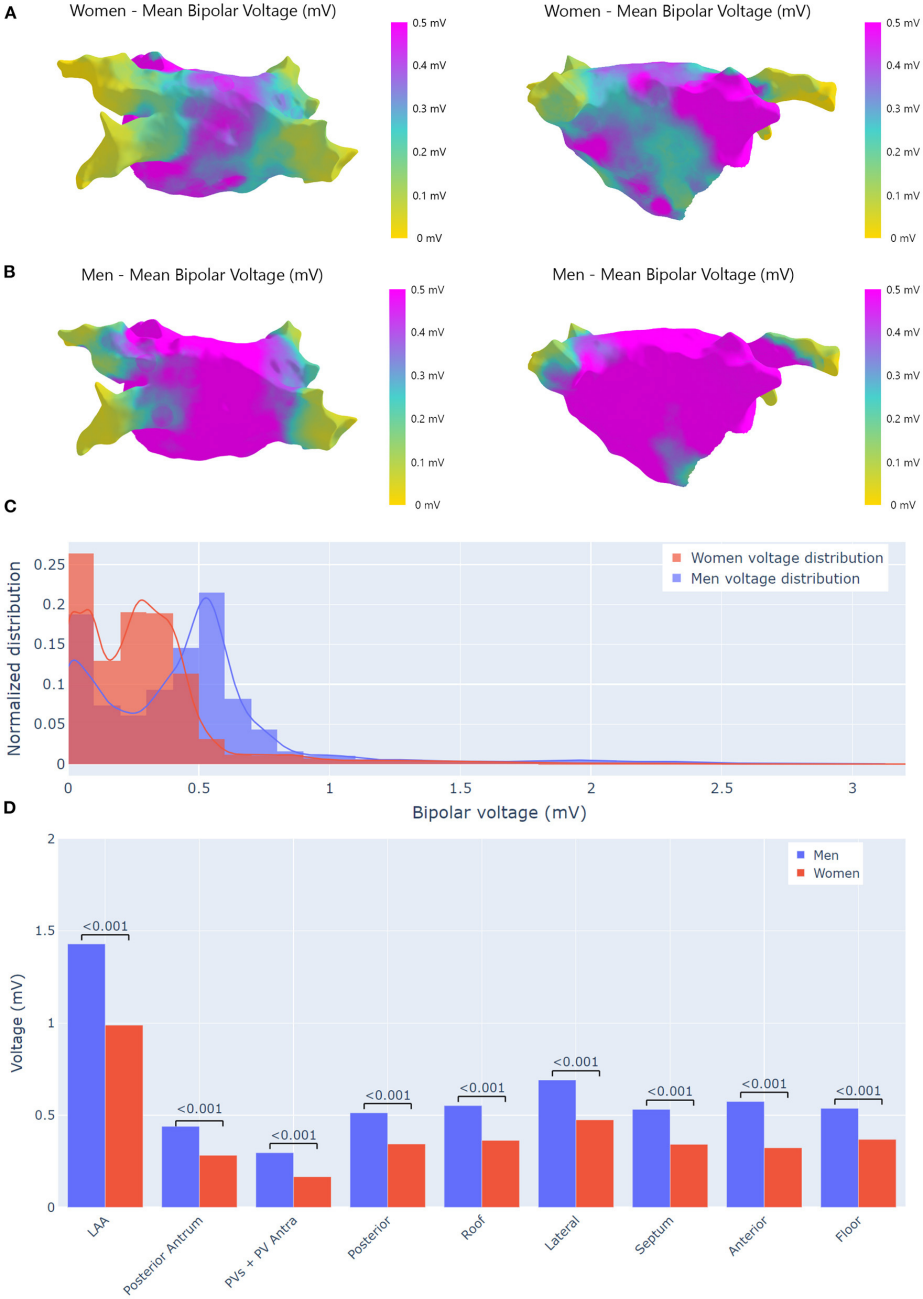
Voltage analysis. **(A)** Bipolar voltage maps differences by gender. Bipolar voltage maps were obtained averaging all recorded points that were projected onto a reference left atrium (LA) mesh. **(A)** Averaged bipolar voltage map from 21 women in which the extension of low-voltage areas is appreciated, the LAA seems to avoid the extension of fibrosis. **(B)** Averaged bipolar voltage map from 64 men where the voltage is preserved in the whole atrium. **(C)** Bipolar voltage points distributions by gender. **(D)** Gender bipolar voltage differences by area. Bipolar voltage was significantly higher in men. In women only, the LAA had a voltage higher than 0.5 mV. LAA, left atrial appendage; PVs, pulmonary veins.

Differences between patients with and without RAc and between men and women are shown in Table 2. Propensity score matching analysis is shown in Supplementary Table 2. The left atrial structural remodeling was assessed by ultra-high-density mapping, 7.952.3 ± 3.325.8 points per map in men and 7.000.1 ± 2.924.2 points per map in women, *p* = 0.219. Structural remodeling in the light of lower voltage and larger low-voltage areas was more advanced in patients with no RAc and women (Figure 2). In women, only the LAA and the lateral wall had an average voltage above the threshold of 0.35 mV which better correlates with delayed enhancement MRI (25).

**Table 2:** Electroanatomical (EA) and AF drivers mapping. RAc and gender differences.

	**Overall**	**No RAc**	**RAc**	***P*** -value^*^	**Men**	**Women**	***P*** -value^*^
N	85 (100.0)	30 (35.3)	55 (64.7)	-	64 (75.3)	21 (24.7)	-
**Electroanatomical mapping**							
Num. EA points	7,717.0 ± 3,240.7	6,985.5 ± 2,961.6	8,116.0 ± 3,341.7	0.113	7,952.3 ± 3,325.8	7,000.1 ± 2,924.2	0.219
Num. cartofinder sites	36.4 ± 14.7	31.1 ± 15.6	39.3 ± 13.5	**0.018**	37.8 ± 15.0	32.3 ± 13.2	0.124
LA volume (*cm*^3^)	148.3 ± 38.7	135.3 ± 34.2	155.4 ± 39.5	**0.017**	153.4 ± 38.5	132.9 ± 36.0	**0.033**
LA area (*cm*^2^)	164.3 ± 31.7	150.8 ± 33.9	171.7 ± 28.0	**0.006**	167.9 ± 32.5	153.4 ± 26.9	**0.049**
LA volume/BSA (*cm*^3^/*m*^2^)	74.0 ± 19.3	69.9 ± 19.2	76.3 ± 19.2	0.146	74.5 ± 19.1	72.6 ± 20.3	0.710
LA area/BSA (*cm*^2^/*m*^2^)	82.0 ± 5.9	77.5 ± 18.0	84.5 ± 14.2	0.070	81.4 ± 16.0	83.3 ± 15.6	0.553
Total procedural time (min)	258 ± 36	241 ± 22	245 ± 41	0.560	260 ± 38	248 ± 33	0.172
Total mapping time (min)	61 ± 18	51 ± 17	66 ± 17	**<0.001**	63 ± 16	56 ± 25	0.239
**Voltage mapping**							
Mean bipolar voltage (mV)	0.6 ± 0.3	0.5 ± 0.2	0.6 ± 0.3	**0.017**	0.6 ± 0.3	0.4 ± 0.2	**<0.001**
LA area <0.5 mV (%)	66.8 ± 20.4	73.5 ± 18.7	63.1 ± 20.6	**0.021**	62.4 ± 19.9	80.0 ± 16.1	**<0.001**
LA area <0.35 mV (%)	52.5 ± 21.3	60.3 ± 21.5	48.2 ± 20.2	**0.015**	47.1 ± 19.0	68.7 ± 19.4	**<0.001**
LA area <0.1 mV (%)	20.3 ± 8.5	21.7 ± 8.0	19.6 ± 8.8	0.270	19.1 ± 7.6	24.2 ± 10.1	**0.044**
**EGM signal analysis**							
EGMs cycle length (ms)	174.9 ± 33.7	188.4 ± 42.4	167.5 ± 25.4	**0.018**	167.4 ± 23.0	197.7 ± 48.7	**0.011**
EGM electrical burden	0.3 ± 0.2	0.2 ± 0.1	0.3 ± 0.2	**0.021**	0.3 ± 0.2	0.2 ± 0.2	**0.029**
**Mechanistic mapping: rotational activity drivers**							
Num. patients with RAc	55	0 (0.0)	55 (100.0)	1.000	47 (73.4)	8 (38.1)	**0.003**
RAc sites per patient with RAc	3.8 ± 3.2	-	3.8 ± 3.2	-	3.9 ± 3.4	3.1 ± 1.4	0.296
RAc events per patient with RAc	82.8 ± 129.4	-	82.8 ± 129.4	-	89.3 ± 138.1	44.5 ± 35.3	0.073
RAc events per acquisition per patient with RAc	2.5 ± 4.4	-	2.5 ± 4.4	-	2.6 ± 4.7	1.8 ± 2.0	0.431
RAc event duration (ms)	586.2 ± 531.9	-	586.2 ± 531.9	-	586.8 ± 550.1	582.0 ± 378.0	0.863
Total RAc event durations per RAc acquisition (ms)	4,361.5 ± 3,536.4	-	4,361.5 ± 3,536.4	-	4,314.8 ± 3,613.6	4,703.3 ± 2,886.8	0.552
Dominant cycle length for RAc acquisitions	161.3 ± 25.5	-	161.3 ± 25.5	-	159.0 ± 24.5	177.8 ± 26.3	**0.002**
**Mechanistic mapping: focal activity drivers**							
Num. patients with FAc	85 (100.0)	30 (100.0)	55 (100.0)	1.000	64 (100.0)	21 (100.0)	1.000
FAc sites per patient with FAc	13.4 ± 9.2	10.4 ± 7.0	15.0 ± 9.9	**0.017**	14.3 ± 9.7	10.6 ± 6.9	0.066
FAc events per patient with FAc	793.6 ± 947.3	486.9 ± 432.8	960.9 ± 1,098.0	**0.007**	886.3 ± 1,037.9	511.0 ± 493.2	**0.032**
FAc events per acquisition per patient with FAc	20.7 ± 19.6	16.1 ± 13.8	23.2 ± 21.7	0.072	22.3 ± 20.8	15.5 ± 14.0	0.102
FAc event duration per FAc acquisitions (ms)	274.6 ± 228.8	293.6 ± 248.7	269.2 ± 222.6	**<0.001**	268.0 ± 217.3	307.8 ± 277.2	**<0.001**
Total FAc event durations per FAc acquisition (ms)	5,743.1 ± 5,907.5	5,135.9 ± 5,436.0	5,961.2 ± 6,053.1	**0.030**	5,803.8 ± 5,978.9	5,497.5 ± 5,602.9	0.473
Dominant cycle length for FAc acquisitions	172.5 ± 29.2	181.4 ± 33.5	169.3 ± 26.7	**<0.001**	169.1 ± 27.0	186.2 ± 33.1	**<0.001**

*Values in the table are n (%) or mean ± SD. AF, atrial fibrillation; EA, Electroanatomical; LA, left atrium; RAc, rotational activity; FA, focal activity; EGM, electrogram*.

* *Categorical data with the chi-square test for categorical data, continuous variables using Welch's two-sample t-test, and proportions based on normal z-test. Bold values indicates statistical significance*.

CartoFinder analyzed 46,425 bipolar EGMs from 3,095 acquisitions (37.8 ± 15.0 per man and 32.3 ± 13.2 per woman) in which the PentaRay splines were orderly deployed. RAc was found in 55 patients (64.7%), with a total of 4,555 RAc events in 208 different CartoFinder acquisitions. Areas with RAc showed a higher voltage than no-RAC areas (0.77 ± 0.46 vs. 0.53 ± 0.37 mV, *p* < 0.001). Revision of bipolar sequences by electrophysiologists confirmed the presence of RAc in all acquisitions in which CartoFinder detected RAc.

**Figure 3:**
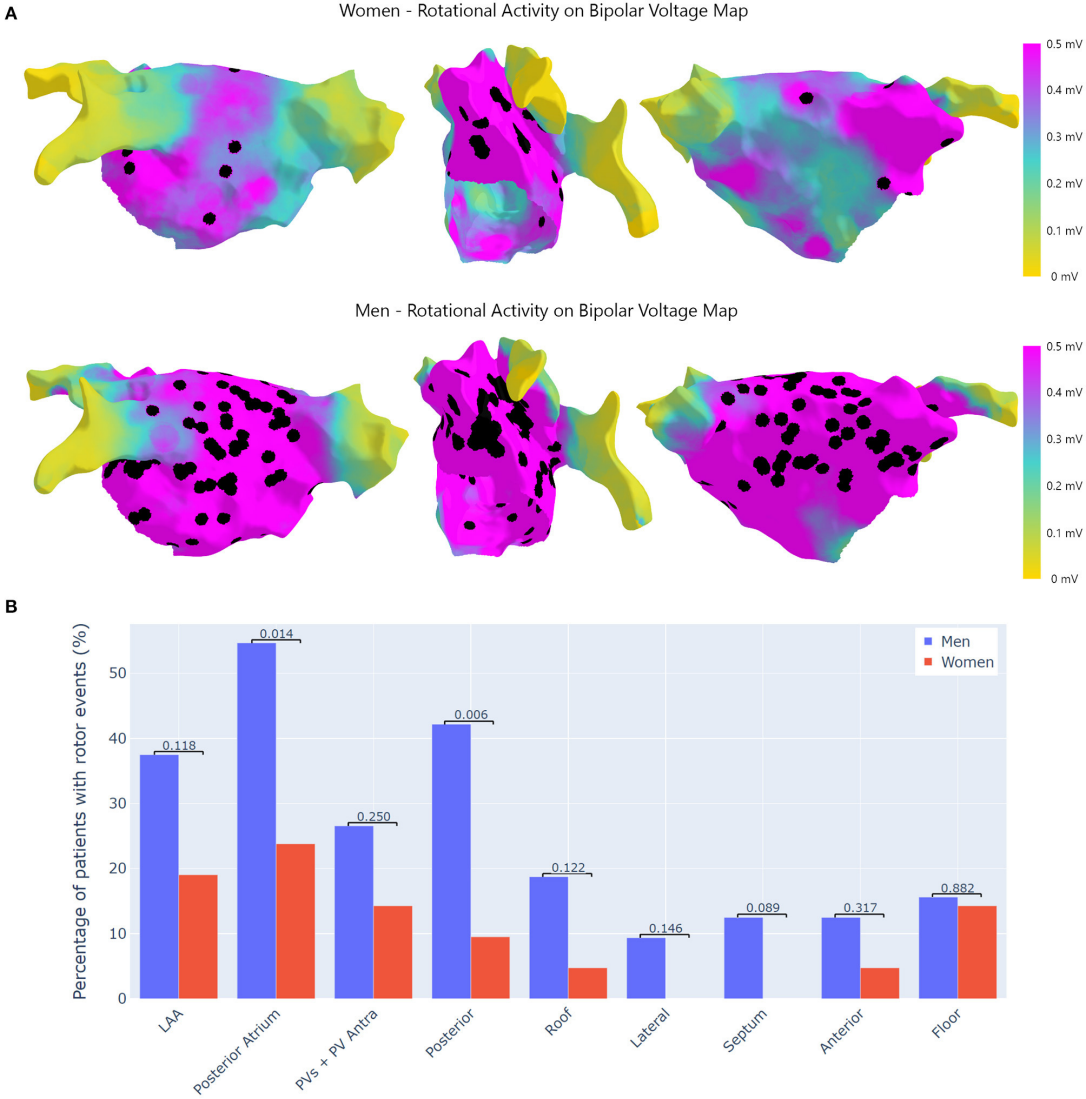
Location of RAc. **(A)** Top: RAc in women. Bottom: RAc in men. RAc was located outside low-voltage areas. This explains why in women RAc converges in and around the LAA. In men as voltage around PV antra is well-preserved RAc is frequently observed. **(B)** Differences in RAc by regions. This figure shows the percentage (%) of patients with RAc at the pre-specified regions of the LA. RAc, rotational activity; LAA, left atrial appendage; PVs, pulmonary veins.

Rotational activity was more frequently recorded in men than women (73.4 vs. 38.1%, *p* = 0.003). The anatomical locations of all rotational events are displayed in Figure 3A. There were gender differences in the anatomical distribution of RAc (Figure 3B), such as the posterior wall (42.0% of men vs. 9.5% of women, *p* = 0.006) and the posterior atrium, such as PVs and posterior wall (54.7% of men vs. and 23.8% of women; *p* = 0.01). The LAA was the area that most frequently hosted RAc events in women.

#### Focal Activity

**Figure 4:**
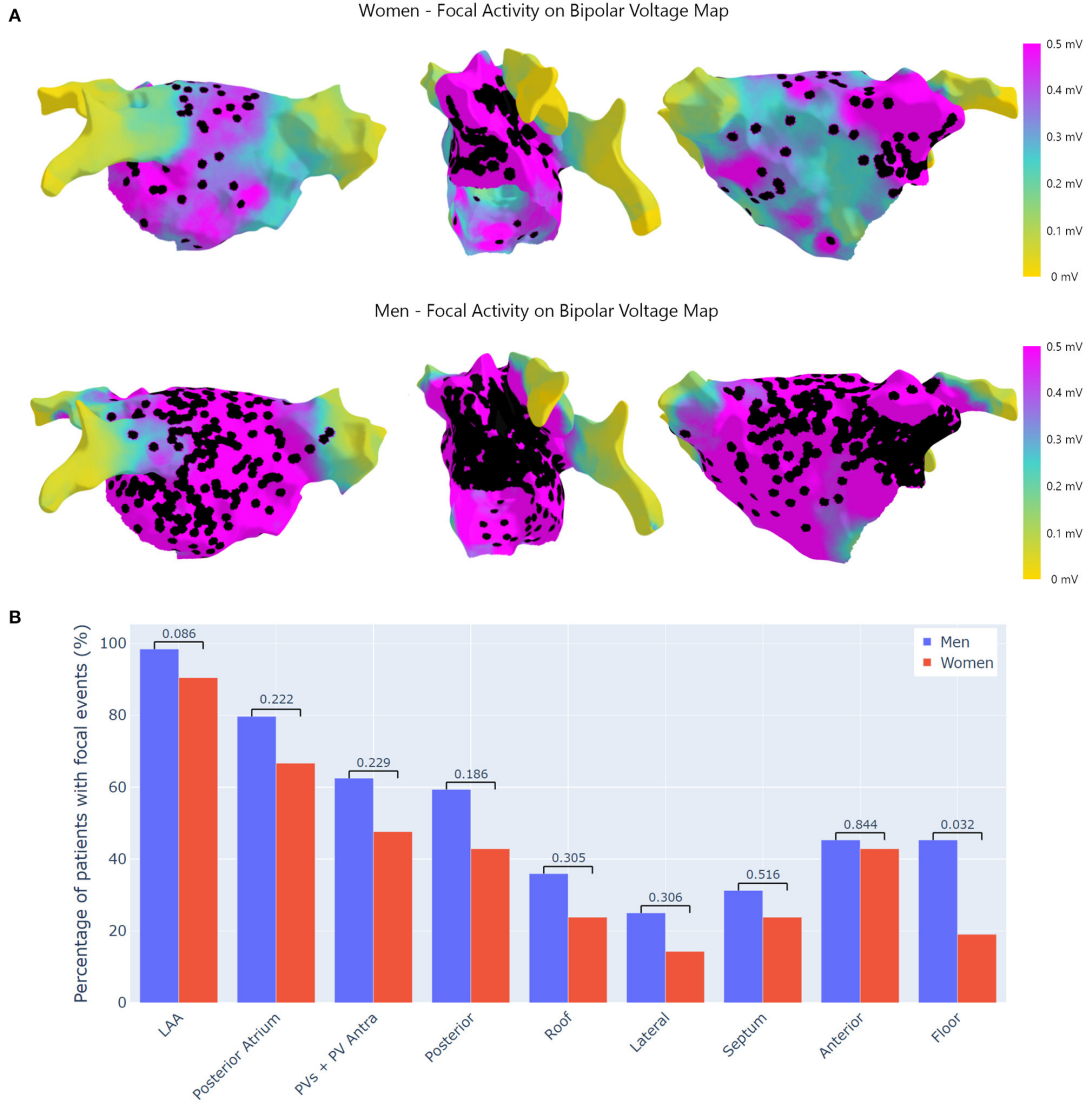
Location of focal activity. **(A)** Top: focal activity in women. Bottom: focal activity in men. Focal activity located in all the LA, with high density predominance in the LAA where it is more frequently detected for all the patients. **(B)** Differences in focal activity by regions. This figure shows the percentage (%) of patients with focal activity at the pre-specified regions of the LA. LAA, left atrial appendage; PVs, pulmonary veins.

The number of focal events was 67.454 (56.723 in men and 10.731 in women), and were present in 1.136 different acquisitions (914 men and 222 women). All patients presented FAc and the number of focal events per patient was significantly higher in men (*p* = 0.032, Table 2). The anatomical distribution of FAc was similar between genders (Figure 4). The area that exhibited more FAc was the LAA for both men and women (98.4 vs. 90.5%, *p* = 0.086).

### Predictors of Post-ablation AF/AT Recurrences

**Figure 5:**
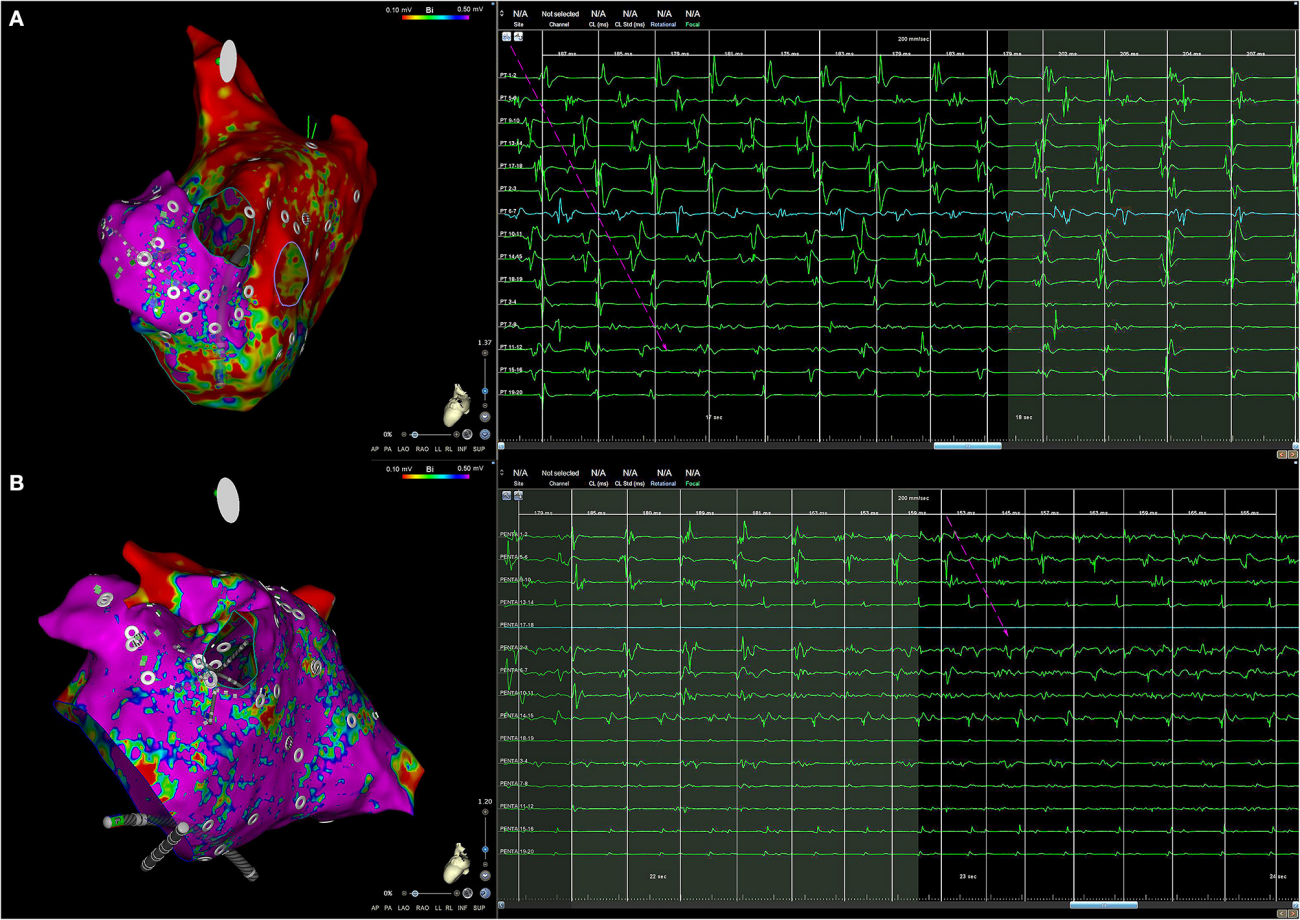
**(A)** Example of a woman with AF recurrence after PVI. Left: RAc was detected only at the LAA, the remaining atrium had low voltage and RAc was not recorded inside the ablation line. Right: the rotational event expands the whole EGM cycle, highlighted with white arrows. The rotational event stops and the cycle length increases with almost simultaneous EGM activations. **(B)** Example of a man with no AF recurrence after PVI. The voltage is almost normal in the whole atrium (left), RAc was detected in the left inferior PV antra (right). The rotational event starts at the same time that acceleration of discrete EGMs appears (Penta 13–14 EGM) and fragmentation in middle ring electrodes (from Penta 3–4 to Penta 11–12 EGMs). AF, atrial fibrillation; PVI, pulmonary vein isolation; LAA, left atrial appendage; EGM, electrogram; PV, pulmonary vein.

Table 3 shows the differences between patients with and without recurrences. Propensity score matching analysis is shown in Supplementary Table 3. The association between AF/AT recurrences and AF drivers was analyzed in 79 patients with a follow-up longer than the 3 months post-ablation blanking period window. After a mean follow-up period of 357 days, AF/AT recurred in 36 patients, 25 men, and 11 women, with a median recurrence of 177.5 days. The LA volume and area were significantly larger in patients with recurrences than those free of AF episodes. Patients with AF/AT recurrences had a higher incidence of RAc, more RAc sites, and higher electrical burden. The differences between patients with and without recurrences regarding the distribution of the RAc in relation to the ablation line during WACPVI are also shown in Table 3. When RAc was only recorded inside the ablation line, most patients were free of recurrences and there was a strong association between RAc outside the ablation and AF/AT recurrences which remained as the main predictor of recurrence after being adjusted for LA volume, surface, and ≤ 0.5 mV area (Table 4 and Figure 5A).

**Table 3:** Atrial fibrillation/Atrial tachycardia (AT) recurrence analysis.

	**Overall Follow-up**	**AF/AT-Free**	**AF/AT Recurrence**	***P*** -value^*^
**N**	79	43 (54.4)	36 (45.6)	
**Gender**
Men	58	33 (56.9)	25 (43.1)	0.634
Women	21	10 (47.6)	11 (52.3)	
**Electroanatomical mapping**
Num. EA points	7,637.2 ± 3265.0	7,405.3 ± 3,047.5	7,914.1 ± 3,531.0	0.500
Num. CartoFinder sites	36.4 ± 15.0	32.1 ± 10.1	41.6 ± 18.1	**0.007**
LA volume (*cm*^3^)	148.2 ± 39.5	134.0 ± 33.1	165.1 ± 40.4	**<0.001**
LA area (*cm*^2^)	164.1 ± 32.5	155.8 ± 24.1	174.0 ± 38.5	**0.017**
**Voltage mapping**
Mean bipolar voltage (mV)	0.6 ± 0.3	0.6 ± 0.3	0.5 ± 0.2	0.114
LA area <0.5 mV (%)	67.2 ± 20.1	63.3 ± 20.1	71.9 ± 19.1	**0.047**
LA area <0.35 mV (%)	53.1 ± 21.2	48.7 ± 20.1	58.3 ± 21.2	**0.046**
LA area <0.1 mV (%)	20.6 ± 8.5	19.2 ± 7.0	22.22 ± 9.8	0.130
**EGM signal analysis**
EGMs cycle length (ms)	174.4 ± 80.6	176.2 ± 84.2	175.6 ± 82.0	0.851
EGM electrical burden	0.3 ± 0.3	0.3 ± 0.2	0.2 ± 0.2	**<0.001**
**Mechanistic mapping: rotational activity drivers**
Num. patients with RAc	50 (63)	23 (53)	27 (75)	**0.048**
RAc sites per patient with RAc	3.7 ± 3.0	2.8 ± 1.8	4.5 ± 3.6	**0.040**
RAc events per patient with RAc	71.2 ± 110.1	57.9 ± 77.8	82.5 ± 130.5	0.424
RAc events per acquisition per patient with RAc	2.0 ± 3.0	1.9 ± 2.8	2.1 ± 3.1	0.799
RAc event duration (ms)	588.9 ± 459.7	591.2 ± 458.9	587.8 ± 460.1	0.887
Total RAc event durations per RAc acquisition (ms)	4,351.4 ± 3529.5	4,212.0 ± 3,363.6	4,425.2 ± 3,612.0	0.692
Dominant cycle length for RAc acquisitions	164.8 ± 24.4	167.0 ± 28.5	163.6 ± 21.8	0.397
**Mechanistic mapping: focal activity drivers**
Num. patients with FA	79	43 (54.4)	36 (45.6)	
FA sites per patient with FA	13.4 ± 9.2	12.8 ± 8.8	14.2 ± 9.6	0.518
FA events per patient with FA	758.3 ± 901.4	640.8 ± 600.8	898.6 ± 1,147.1	0.236
FA events per acquisition per patient with FA	19.7 ± 18.1	19.4 ± 16.1	20.1 ± 20.3	0.870
FA event duration per FA acquisitions (ms)	276.8 ± 230.2	272.1 ± 222.2	281.1 ± 237.1	**<0.001**
Total FA event durations per FA acquisition (ms)	5,653.7 ± 5816.1	5,419.9 ± 5,544.3	5,898.5 ± 6,078.0	0.185
Dominant cycle length for FA acquisitions	173.5 ± 28.9	174.9 ± 30.3	172.1 ± 27.2	0.108
**RAc presence location**
No RAc	29 (36.7)	20 (69.0)	9 (31.0)	**0.002**
RAc only inside WACPVI	9 (11.4)	8 (88.9)	1 (11.1)	
RAc outside WACPVI	41 (51.9)	15 (36.6)	26 (63.4)	

*Values in the table are n (%) or mean ± SD. AF, atrial fibrillation; AT, atrial tachycardia; RAc, rotational activity; WACPVI, wide area circumferential pulmonary vein isolation*.

* *Categorical data with the chi-square test for categorical data, continuous variables using Welch's two-sample t-test, and proportions based on normal z-test. Bold values indicates statistical significance*.

**Table 4:** Predictors of AF recurrence analysis after WACPVI ablation.

	**Univariate analysis**		**Multivariate analysis**	
	**OR (95% CI)**	***P*** **-value**	**OR (95% CI)**	***P*** **-value**
RAc outside WACPVI	4.2 (1.6–11.2)	**0.002**	4.4 (1.5–13.8)	**0.007**
Scar (<0.5 mV)	5.4 (0.9–35.3)	**0.06**	10.2 (1.3–91)	0.1
LA area (*cm*^2^)	83 (2.7–4,183)	**0.01**	13.0 (0.4–776)	0.8
LA volume (*cm*^3^)	69.9 (6.3–1,036.7)	**0.001**	32.0 (0.8–6462)	0.08

*RAc, rotational activity; WACPVI, wide area circumferential pulmonary vein isolation; LA, left atrium; OR, odds ratio; CI, confidence interval. Bold values indicates statistical significance*.

## Discussion

### Main Findings

This study describes the relationship between structural remodeling and AF drivers that could explain some gender differences in patients with persistent AF. First, RAc distribution in the LA seems related to the degree of structural remodeling which at the ablation time is more intense in women despite having a similar AF duration. Second, consequently with previous findings, women in comparison with men have much less RAc which is commonly located outside the PV antra. Third, post-ablation AF recurrence was associated with RAc outside WACPVI.

### Structural Remodeling

Voltage mapping was performed during AF based on previous data in which the correlation between low-voltage and contrast-enhanced MRI is significantly better when EGMs are acquired during AF than during sinus rhythm (25). Structural changes in LA in women are more extensive as suggested by the lower average voltage and larger scar areas. Since AF duration was similar, these changes could be due to the fact that women were older and had more hypertension. Nevertheless, the analysis based on propensity score matching suggests that differences in remodeling are not explained by differences in risk factors alone, but are also due to a gender effect. A previous study has revealed that women with longstanding persistent AF had a higher degree of fibrosis that could be due to the inherent differential expression of fibrosis-related genes (7). Voltage mapping suggests that in women fibrosis affects the posterior atrium more significantly than the LAA, which seems to resist the advance of fibrosis (Figure 2).

### Rotational Activity

With the use of new mapping techniques, several studies have suggested that rotors are a part of the electrophysiological AF substrate (26), but controversy remains over whether the RAc is critical for the maintenance of AF or if it is merely due to the collision of wandering atrial activation wave-fronts (27).

CartoFinder software has been reported to identify with high reproducibility RAc during both paroxysmal and persistent AF when applied to basket catheter recordings (17). In this study, we applied the CartoFinder software to endocardial recordings obtained with a high-resolution catheter that allows assessing the activation sequence with high-quality bipolar EGMs in which far-field activity is canceled. The presence of RAc was not only based on signal processing since all CartoFinder acquisitions were revised by electrophysiologists who confirmed the RAc when the sequential activation spanned the whole cycle length and it coincided with the appearance of high-frequency low amplitude at the inner rings and the acceleration of discrete EGMs (Figure 5). The fact that high-resolution catheters were used to detect RAc and assess structural remodeling reinforces the relationship between structural remodeling and the anatomical distribution of RAc.

The incidence of RAc was lower than in previous studies in which RAc was detected based on the signal processing of unipolar EGMs (15, 28, 29). It could be that high-resolution mapping was more specific to detect RAc. Inaccuracies during rotor identification may explain why the Randomized Evaluation of Atrial Fibrillation Treatment With Focal Impulse and Rotor Modulation Guided Procedures (REAFFIRM) trial found no difference between rotor ablation and conventional ablation (30).

We have not observed RAc in low voltage areas which is consistent with prior studies (26). Computational studies demonstrated that rotational drivers perpetuating AF were localized in boundary zones between fibrotic and non-fibrotic tissue (31) and that the moderate levels of fibrotic tissue, i.e., 40% of fibrotic elements, can anchor re-entry activity (32). Nevertheless, the higher levels of fibrosis can block the fast propagation of the electrical wavefronts since the interactions between myocytes are reduced (33). Low voltage areas probably (because of extensive fibrosis) may have more difficulties for housing a functional re-entry, this explains the lower incidence of RAc in the women group and suggests that RAc is not the main mechanism maintaining persistent AF in women and patients with extensive fibrosis.

### RAc and WACPVI Efficacy

Although this study did not probe RAc as a mechanism that maintains persistent AF, the association between the presence and dispersion of RAc and the post-ablation AF recurrences highlights the role of RAc in AF. RAc outside the ablation area was the main predictor of AF recurrence and when RAc was included in the ablation line recurrences were very low, suggesting that AF drivers were eliminated. However, the absence of RAc outside WACPVI did not ensure freedom from recurrence, especially in women, suggesting that there are unidentified mechanisms other than RAc involved in AF recurrence after ablation.

### Study Limitations

This was a retrospective single cohort and single-center study. The proportion of women and men in this study was not balanced possibly representing the real practice in which women are underrepresented in catheter ablation series (34). In the study, we included 69 first ablation and 16 redo procedures, equally distributed among men and women (*p* = 0.542). Patients in sinus rhythm in whom stable fibrillation was not induced to allow mapping of the RAc were not included. The right atrium was not explored and remapping was not done after ablation. Ablation was not guided by RAc. We did not quantify the inter-intraobserver correlation of RAc.

## Conclusion

In women, structural remodeling at the ablation time is more severe and affects more intensively the posterior atrium. Structural remodeling could determine RAc distribution. RAc outside the ablation line is the main predictor of AF/AT recurrence suggesting RAc is mechanistically involved in AF maintenance.

## Data Availability Statement

The data of this study are available from the corresponding authors on request.

## Ethics Statement

The studies involving human participants were reviewed and approved by Local Ethics Committee of the institution and following the European and Spanish regulations on the subject (Code: GÉNERO-FA, Approval date: 8th March 2021). The Ethics Committee waived the requirement of written informed consent for participation.

## Author Contributions

PÁ, AC, FA, TD, EG-T, and FF-A collected data and gave final approval of the manuscript. ÁA, NS, and GR-M conceived and designed the study, analyzed the data, drafted the manuscript, and gave final approval of the manuscript. All authors substantially contributed to conducting the underlying research and drafting the manuscript.

## Funding

This study was supported by the Instituto de Salud Carlos III, Madrid, Spain (PI18/01895 and DTS21/00064), Red de Terapia Celular from the Instituto de Salud Carlos III, Madrid, Spain (RD16/0011/0029), Ricors—Red de Investigación Cooperativa Orientada a Resultados en Salud – RICORS TERAV (RD21/0017/0002), and the Sección del Ritmo de la Sociedad Española de Cardiología (Grant: Beca de la Asociacion del Ritmo para formación en investigacion post-residencia en centros españoles de la Sección del Ritmo de la Sociedad Española de Cardiología), Madrid, Spain.

## Conflict of Interest

PÁ received teaching honoraria from Medtronic and served as an Advisory Board member for Boston Scientific. FA served as an Advisory Board member for Medtronic and MicroPort. TD received teaching honoraria from Medtronic. ÁA is a consultant for Medtronic and Boston Scientific. The remaining authors declare that the research was conducted in the absence of any commercial or financial relationships that could be construed as a potential conflict of interest.

## Publisher's Note

All claims expressed in this article are solely those of the authors and do not necessarily represent those of their affiliated organizations, or those of the publisher, the editors and the reviewers. Any product that may be evaluated in this article, or claim that may be made by its manufacturer, is not guaranteed or endorsed by the publisher.
