# Supplementary material for: Structural Remodeling and Rotational Activity in Persistent/Long-Lasting Atrial Fibrillation: Gender-Effect Differences and Impact on Post-ablation Outcome
Source: Front Cardiovasc Med. 2022 Mar 21;9:819429. doi: 10.3389/fcvm.2022.819429 (PMC8977980; doi:10.3389/fcvm.2022.819429)
Supplement: Supplementary file 1 [file Data_Sheet_1.PDF]

Supplementary Material

1 Supplementary Methods

1.1 EGM Electrical Burden detection

For a given electrogram (EGM) signal, i.e., $x[n]$ where n denotes the *n-th* acquisition time instant of a total signal length of N samples, a binary signal $b[n]$ is obtained. This signal is computed using the method described in (1). The method automatically detects periods of electrical activity in atrial fibrillation (AF) bipolar EGMs. For electrically silent periods in the EGM, the method sets the binary signal $b[n] = 0$, and $b[n] = 1$ for electrically active segments. Therefore, for one EGM signal, the overall electrical burden (EB) is calculated as follows, $EB = \frac{1}{N} \sum_{1}^{N} b[n]$, where $EB \in [0,1]$. For a sampling frequency $fs = 1kHz$ and 30-second signals, the number of samples is $N = 30000$ samples. Signals with higher continuous fragmentation presence will have higher EB, while EGM with longer isoelectric lines or no activity will have lower EB values.

1.2 Supplementary Figures

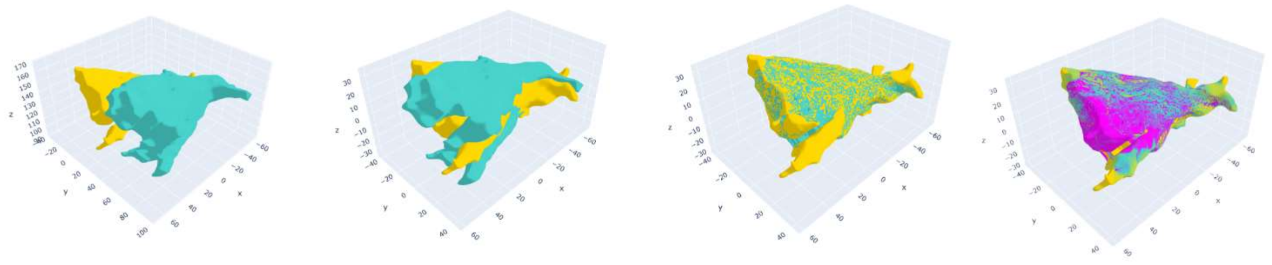

A B C D

Supplementary Figure 1. Atrial 3D merge. A. Two atrial meshes, the reference (target) mesh in yellow, and in blue the mesh of a patient (source) in blue to be merged into the reference one. B. Pre-alignment of the reference and source meshes by mean coordinate subtraction. C. Non-rigid ICP merging method projects the mesh of the patient onto the reference mesh. D. Final voltage projection from the source mesh onto the target mesh. ICP, iterative closest point.

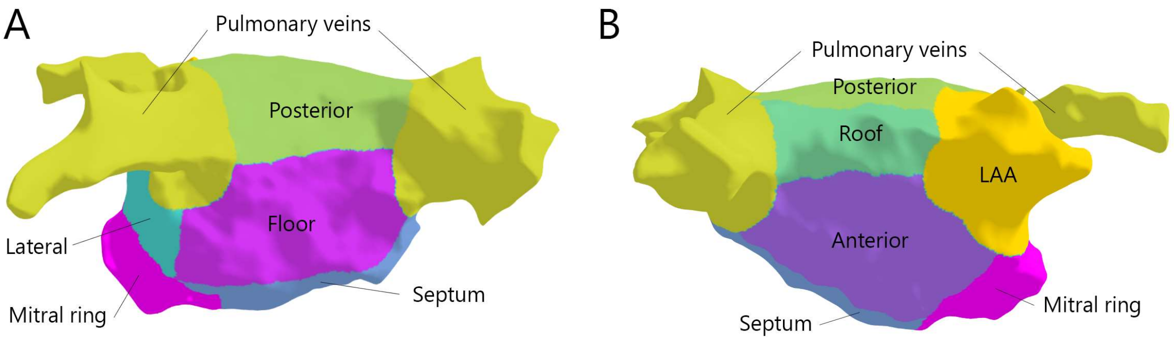

Supplementary Figure 2. Left atrium segmentation. A. Posterior view of the left atrium. B. Anterior view of the left atrium. Segmented regions: LAA, pulmonary veins, posterior wall, atrial roof, lateral wall, septum, anterior wall, atrial floor, and mitral ring. LA, left atrium; LAA, left atrial appendage.

2 Propensity Score Analysis

A propensity score matching was applied to reduce the effect of baseline differences in the data population by gender. We ran a logistic regression on the gender group and as covariates for the analysis, we selected those that were significantly different ($p<0.05$) in Table 1, i.e., age, hypertension, BSA, and CHA2DS2-VASc. The propensity variable obtained matched 21 women to 16 men with similar characteristics to obtain a trustful baseline comparison between gender groups. We present the propensity score matched differences in Supplementary Table 1. After

The sex difference bias effect was also compensated by completing the same statistical analysis for Table 2 containing the electroanatomical information of the patients for presence of rotational activity and gender groups, and also for the results of Table 3 for the AF/AT recurrence groups. The propensity-matched analysis for both tables is shown in Supplementary Table 2 and Suppelementary Table 3 respectively.

The logistic regression information for the age, hypertension, BSA, and CHA2DS2-VASc is included in Supplementary Table 4. The main variables of a typical logistic regression model were included.

3 Supplementary References

1. Ríos-Muñoz GR, Moreno-Pino F, Soto N, Olmos PM, Artés-Rodríguez A, Fernández-Avilés F, et al. Hidden Markov Models for Activity Detection in Atrial Fibrillation Electrograms. 2020 Comput. Cardiol. Conf., 2020, p. 1–4. doi:10.22489/CinC.2020.098.

4 Supplementary Tables

Supplementary Table 1. Baseline characteristics of the patients after propensity score matching for gender and age, hypertension, BSA, and CHA2DS2-VASc covariates. Rotational activity and gender differences.

		Overall	Men	Women	P-value*
**N**		37.0(100.0)	16.0(43.2)	21.0(56.8)	-
**Age (years)**		63.5±9.2	61.3±9.5	65.2±8.6	0.218
**Procedure Number**		1.2±0.4	1.1±0.3	1.2±0.5	0.644
**Comorbidities**					
**Heart failure**		5.0(13.5)	1.0(6.3)	4.0(19.0)	0.478
**Hypertension**		24.0(64.9)	9.0(56.3)	15.0(71.4)	0.541
**Diabetes mellitus**		8.0(21.6)	2.0(12.5)	6.0(28.6)	0.423
**Dyslipidemia**		18.0(48.6)	8.0(50.0)	10.0(47.6)	0.851
**COPD**		2.0(5.4)	2.0(12.5)	0.0(0.0)	0.180
**Obstructive sleep apnea**		6.0(16.2)	4.0(25.0)	2.0(9.5)	0.371
**Stroke**		5.0(13.5)	1.0(6.3)	2.0(9.5)	1.000
**SHD**		8.0(21.6)	3.0(18.8)	5.0(23.8)	1.000
**BSA (m^2)**		1.9±0.1	1.9±0.1	1.8±0.1	0.238
**CHA2DS2-VASc**		1.9±1.3	1.6±0.4	2.1±0.0	0.234
**New York Heart Association Functional Classification**		3.0(8.1)	0.0(0.0)	3.0(14.3)	0.114
	**I**	10.0(27.0)	7.0(43.8)	3.0(14.3)	
	**II**	15.0(40.5)	7.0(43.8)	8.0(38.1)	
	**III**	9.0(24.3)	2.0(12.5)	7.0(33.3)	
	**IV**	0.0(0.0)	0.0(0.0)	0.0(0.0)	
**Diagnosis of AF (years)**		2.6±2.7	2.6±2.7	2.6±2.8	
**Echocardiographic parameters**					
**LVEF (%)**		57.9±10.1	58.1±10.9	57.7±9.5	0.927
**LA area (cm^2)**		25.6±7.6	25.2±10.1	25.9±4.5	0.861
**LA area/BSA (cm^2/m^2)**		13.8±3.8	13.2±4.8	14.4±2.5	0.493

Values in the table are n (%) or mean±standard deviation (SD). COPD, chronic obstructive pulmonary disease; SHD, structural heart disease; BSA, body surface area; NYHA, New York Heart Association; LVEF, left ventricular ejection fraction; and LA, left atrium. *Categorical data with the Chi-square test for categorical data, continuous variables using Welch's two-sample t-test, and proportions based on normal z-test.

frontiers

Supplementary Table 2. Electroanatomical and AF drivers mapping after propensity score matching for gender and age, hypertension, BSA, and CHA2DS2-VASc covariates. Rotational activity and gender differences.

	Overall	No RAc	RAc	P-value*	Men	Women	P-value*
N	37.0(100.0)	17.0(45.9)	20.0(54.1)	-	16.0(43.2)	21.0(56.8)	-
**Electroanatomical Mapping**							
**Num. EA points**	7478.7±3413.3	6976.0±3358.9	7906.1±3401.0	0.423	8106.9±3944.5	7000.1±2924.2	0.366
**Num. CartoFinder sites**	33.7±13.5	29.2±8.7	37.6±15.5	0.053	35.5±14.0	32.3±13.2	0.498
**LA volume (cm^3)**	132.0±37.4	128.5±38.1	135.1±36.5	0.609	130.9±40.1	132.9±36.0	0.873
**LA area (cm^2)**	150.4±34.3	139.9±38.3	159.4±27.5	0.101	146.4±42.3	153.4±26.9	0.578
**LA volume/BSA (cm^3/m^2)**	71.4±21.4	70.5±22.5	72.2±20.4	0.815	69.9±23.3	72.6±20.3	0.714
**LA area/BSA (cm^2/m^2)**	81.0±19.2	76.3±21.7	85.1±15.6	0.188	77.4±22.9	83.3±15.6	0.359
**Total procedural time (min)**	254.0±37.0	233.0±19.0	272.0±40.0	<0.001	263.0±42.0	248±33	0.219
**Total mapping time (min)**	60.0±22.0	46.0±17.0	72.0±18.0	<0.001	65.0±16.0	56.0±25.0	0.192
**Voltage Mapping**							
**Mean bipolar voltage (mV)**	0.5±0.3	0.4±0.2	0.6±0.3	0.052	0.7±0.3	0.4±0.2	**0.007**
**LA area <0.5 mV (%)**	70.4±21.1	77.7±19.4	64.3±20.5	0.002	57.9±20.7	80.0±16.1	**0.002**
**LA area <0.35 mV (%)**	57.7±24.2	66.3±23.8	50.3±23.3	0.047	43.1±22.1	68.7±19.4	**<0.001**
**LA area <0.31 mV (%)**	53.8±23.8	62.1±23.4	46.7±23.1	0.053	39.6±21.6	64.5±19.4	**<0.001**
**LA area <0.1 mV (%)**	21.0±9.9	22.1±8.0	20.1±11.3	0.540	16.8±8.4	24.2±10.1	**0.023**
**EGM Signal Analysis**							
**EGMs cycle length (ms)**	184.3±41.2	198.2±49.7	172.6±27.1	0.079	166.8±20.5	197.7±48.7	**0.014**
**EGM electrical burden**	0.2±0.2	0.2±0.1	0.3±0.2	0.022	0.3±0.2	0.2±0.2	0.116
**Mechanistic Mapping: Rotational Activity Drivers**							
**Num. patients with RAc**	20.0(54.1)	0.0(0.0)	20.0(100.0)	1	12.0(75.0)	8.0(38.1)	**0.026**
**RAc sites per patient with RAc**	3.1±2.4	-	3.1±2.4	-	3.0±2.9	3.1±1.4	0.905
**RAc events per patient with RAc**	34.7±33.9	-	34.7±33.9	-	28.1±31.2	44.5±35.3	0.332
**RAc events per acquisition per patient with RAc**	1.2±1.5	-	1.2±1.5	-	0.9±0.9	1.8±2.0	0.288
**RAc event duration (ms)**	575.0±458.0	-	575.0±458.0	-	569.1±515.2	582.0±378.0	0.744
**Total RAc event durations per RAc acquisition (ms)**	3668.0±1934.6	-	3668.0±1934.6	-	3243.8±1420.7	4703.3±2886.8	0.311
**Dominant cycle length for RAc acquisitions**	173.2±24.9	-	173.2±24.9	-	169.6±24.8	177.8±26.3	0.463
**Mechanistic Mapping: Focal Activity Drivers**							
**Num. patients with FAc**	37.0(100.0)	17.0(100.0)	20.0(100.0)	1	16.0(100.0)	21.0(100.0)	1
**FAc sites per patient with FAc**	11.2±7.2	9.2±5.1	13.0±8.2	0.102	12.1±7.5	10.6±6.9	0.535
**FAc events per patient with FAc**	587.8±620.9	431.1±439.8	721.0±714.3	0.152	688.6±744.6	511.0±493.2	0.431
**FAc events per acquisition per patient with FAc**	17.3±16.1	14.4±14.1	19.8±17.2	0.313	19.6±18.1	15.5±14.0	0.477
**FAc event duration per FAc acquisitions (ms)**	290.5±263.9	302.7±273.9	283.9±258.0	<0.001	273.2±248.5	307.8±277.2	**<0.001**
**Total FAc event durations per FAc acquisition (ms)**	4993.4±2393.3	4555.2±2381.7	5365.8±2339.4	0.319	5052.6±2447.2	5497.5±5602.9	0.900
**Dominant cycle length for FAc acquisitions**	179.1±30.0	187.1±33.1	172.4±25.3	0.154	168.4±21.5	186.2±33.1	**0.049**

Values in the table are n (%) or mean ± standard deviation (SD). AF, atrial fibrillation; EA, Electroanatomical; LA, left atrium; RAc, rotational activity; FA, focal activity; EGM, electrogram. *Categorical data with the Chi-square test for categorical data, continuous variables using Welch's two-sample t-test, and proportions based on normal z-test.

Supplementary Table 3. Atrial fibrillation/Atrial tachycardia recurrence analysis after propensity score matching for gender and age, hypertension, BSA, and CHA2DS2-VASc covariates.

	Overall Follow-up	AF/AT-Free	AF/AT Recurrence	P-value*
**N**	37.0(100.0)	21.0(56.8)	16.0(43.2)	
**Gender**				
**Men**	16.0(43.2)	11.0(68.8)	5.0(31.3)	0.176
**Women**	21.0(56.8)	10.0(47.6)	11.0(52.4)	
**Electroanatomical Mapping**				
**Num. EA points**	7478.7±3413.3	7656.3±3351.4	7245.6±3479.2	0.728
**Num. CartoFinder sites**	33.7±13.5	30.7±8.5	37.6±17.3	0.169
**LA volume (cm^3)**	132.0±37.4	121.2±35.6	146.3±34.8	**0.045**
**LA area (cm^2)**	150.4±34.3	147.0±27.2	154.9±41.4	0.530
**Voltage Mapping**				
**Mean bipolar voltage (mV)**	0.5±0.3	0.6±0.2	0.5±0.3	0.495
**LA area <0.5 mV (%)**	70.4±21.1	67.7±21.1	74.0±20.5	**<0.001**
**LA area <0.35 mV (%)**	57.7±24.2	53.8±23.0	62.8±24.8	**<0.001**
**LA area <0.31 mV (%)**	53.8±23.8	49.7±22.1	59.1±25.0	**<0.001**
**LA area <0.1 mV (%)**	21.0±9.9	19.0±7.2	23.6±12.2	**<0.001**
**EGM Signal Analysis**				
**EGMs cycle length (ms)**	184.3±41.2	188.9±46.8	178.4±31.4	0.437
**EGM electrical burden**	0.2±0.2	0.3±0.2	0.2±0.2	0.638
**Mechanistic Mapping: Rotational Activity Drivers**				
**Num. patients with RAc**	20.0(54.1)	10.0(47.6)	10.0(62.5)	0.549
**RAc sites per patient with RAc**	3.1±2.4	2.4±1.2	3.7±3.1	0.264
**RAc events per patient with RAc**	34.7±33.9	33.3±31.6	36.0±36.0	0.868
**RAc events per acquisition per patient with RAc**	1.2±1.5	1.3±1.5	1.2±1.6	0.884
**RAc event duration (ms)**	575.0±458.0	656.6±603.0	520.3±315.0	**0.003**
**Total RAc event durations per RAc acquisition (ms)**	4034.6±3251.9	4666.4±4010.7	3624.8±2563.8	0.275
**Dominant cycle length for RAc acquisitions**	170.5±27.1	175.3±33.2	167.5±21.6	0.323
**Mechanistic Mapping: Focal Activity Drivers**				
**Num. patients with FA**	37.0(100.0)	21.0(56.8)	16.0(43.2)	
**FA sites per patient with FA**	11.2±7.2	10.9±5.4	11.7±9.0	0.768
**FA events per patient with FA**	587.8±620.9	517.6±428.5	679.9±797.3	0.482
**FA events per acquisition per patient with FA**	17.3±16.1	16.6±13.2	18.1±19.2	0.798
**FA event duration per FA acquisitions (ms)**	290.5±263.9	298.9±263.8	281.8±263.6	**<0.001**
**Total FA event durations per FA acquisition (ms)**	4993.4±2393.3	5150.8±2490.6	4786.8±2242.4	0.653
**Dominant cycle length for FA acquisitions**	179.1±30.0	181.8±31.7	175.6±27.3	0.540
**RAc Presence Location**				
**No RAc**	17.0(45.9)	11.0(64.7)	6.0(35.3)	**0.050**
**RAc only inside WACPVI**	5.0(13.5)	4.0(80.0)	1.0(20.0)	
**RAc outside WACPVI**	15.0(40.5)	6.0(40.0)	9.0(60.0)	

Values in the table are n (%) or mean ± standard deviation (SD). AF, atrial fibrillation; AT, atrial tachycardia; RAc, rotational activity; WACPVI, wide area circumferential pulmonary vein isolation. *Categorical data with the Chi-square test for categorical data, continuous variables using Welch's two-sample t-test, and proportions based on normal z-test.

Supplementary Table 4. Logistic regression coefficients and variables for the propensity score match.

	Variables in the equation					
	**B**	**S.E.**	**Wald χ^2**	**df**	**P-value**	**Exp(B)**
**Age**	0.031	0.043	0.526	1	0.468	1.032
**Hipertension Binary**	1.727	0.766	50.082	1	0.024	5.622
**BSA (m^2)**	-9.191	2.680	110.759	1	0.001	0.000
**CHA2DS2-VASc**	-0.452	0.323	10.958	1	0.162	0.636
**Constant**	14.606	6.112	50.711	1	0.017	22054870.280

B is the regression weight; S.E. is the standard error around the coefficient for the constant; Wald χ^2 is the chi-square test statistic for each predictor variable; df, degrees of freedom of the Wald chi-square test; Exp(B), the exponential value of the B coefficient; BSA, body surface area.
